# Identification and validation of quantitative trait loci for kernel traits in common wheat (*Triticum aestivum* L.)

**DOI:** 10.1186/s12870-020-02661-4

**Published:** 2020-11-23

**Authors:** Hong Liu, Xiaotao Zhang, Yunfeng Xu, Feifei Ma, Jinpeng Zhang, Yanwei Cao, Lihui Li, Diaoguo An

**Affiliations:** 1 Center for Agricultural Resources Research, Institute of Genetics and Developmental Biology, Chinese Academy of Sciences, Shijiazhuang, 050021 China; 2 The National Key Facility for Crop Gene Resources and Genetic Improvement, Institute of Crop Science, Chinese Academy of Agricultural Sciences, Beijing, 100081 China; 3 The Innovation Academy for Seed Design, Chinese Academy of Sciences, Beijing, 100101 China; 4 University of Chinese Academy of Sciences, Beijing, 100049 China

**Keywords:** Kernel traits, Quantitative trait locus, *TaFT-D1*, KASP marker, *Triticum aestivum*

## Abstract

**Background:**

Kernel weight and morphology are important traits affecting cereal yields and quality. Dissecting the genetic basis of thousand kernel weight (TKW) and its related traits is an effective method to improve wheat yield.

**Results:**

In this study, we performed quantitative trait loci (QTL) analysis using recombinant inbred lines derived from the cross ‘PuBing3228 × Gao8901’ (PG-RIL) to dissect the genetic basis of kernel traits. A total of 17 stable QTLs related to kernel traits were identified, notably, two stable QTLs *QTkw.cas-1A.2* and *QTkw.cas-4A* explained the largest portion of the phenotypic variance for TKW and kernel length (KL), and the other two stable QTLs *QTkw.cas-6A.1* and *QTkw.cas-7D.2* contributed more effects on kernel width (KW). Conditional QTL analysis revealed that the stable QTLs for TKW were mainly affected by KW. The QTLs *QTkw.cas-7D.2* and *QKw.cas-7D.1* associated with TKW and KW were delimited to the physical interval of approximately 3.82 Mb harboring 47 candidate genes. Among them, the candidate gene *TaFT-D1* had a 1 bp insertions/deletion (InDel) within the third exon, which might be the reason for diversity in TKW and KW between the two parents. A Kompetitive Allele-Specific PCR (KASP) marker of *TaFT-D1* allele was developed and verified by PG-RIL and a natural population consisted of 141 cultivar/lines. It was found that the favorable *TaFT-D1 (G)-allele* has been positively selected during Chinese wheat breeding. Thus, these results can be used for further positional cloning and marker-assisted selection in wheat breeding programs.

**Conclusions:**

Seventeen stable QTLs related to kernel traits were identified. The stable QTLs for thousand kernel weight were mainly affected by kernel width. *TaFT-D1* could be the candidate gene for QTLs *QTkw.cas-7D.2* and *QKw.cas-7D.1*.

## Background

Common wheat (*Triticum aestivum* L.) is one of the most important cereal crops for feeds 40% of population in the world (http://www.fao.org/). Wheat yield is determined by thousand kernel weight (TKW), kernel number per spike, and effective tiller number [1]. Among them, TKW is the most stable and highest heritable trait, and it is also an important selection target for the genetic improvement of wheat yield [2]. Kernel weight is a complex yield component, which is mainly affected by kernel length (KL), kernel width (KW), kernel length / kernel width (KL/W) and kernel thickness [3]. Therefore, exploring the genetic variation of TKW and its related traits is an effective approach to increase wheat yield [4].

A large number of genes related to kernel weight and morphological traits have been identified in crop. For instance, in rice, *GS3*, *qGL3*, *GL4* and *GLW7* were associated with kernel weight, *GW2*, *GW5*, *GS5* and *GW8* were associated with kernel width [5–12]. Recently, several genes associated with kernel weight have been identified in wheat through comparative genomics approaches, thereby providing an in-depth understanding of the molecular basis of TKW. For example, *TaGW2* and *TaDA1*, which encode an E3 RING ligase [13–15] and a ubiquitin receptor [16], respectively. Both of them are conserved component of the ubiquitin-proteasome pathway and negatively regulate wheat kernel size. In addition, *TaGS5-3A* [17] and *TaFlo2-A1* [18], which encode a serine carboxypeptidase and a protein containing tetratricopeptide repeat motif, respectively, both can regulate kernel size and weight. Genes involved in starch and sucrose metabolism pathways also affect wheat kernel size, such as the cell wall invertase *TaCwi-A1* [19], the sucrose synthases *TaSus1* and *TaSus2* [20], ADP*-*glucose pyrophosphorylase *TaAGP-S1-7A* and *TaAGP-L-1B* [21].

Previous researches have shown that conditional QTL mapping has been used to study genetic basis of complex traits in crops [22, 23]. In wheat, conditional QTL analysis were carried out to evaluate the static genetic control of traits at different growth stages for kernel size and weight [23, 24] and yield [25]; to reveal the dynamic genetic factors of plant height [26, 27]; and to reveal the genetic contribution of different nitrogen and phosphorus supplement environments factors to QTL expression by dissecting QTLs based on trait values conditioned [28].

Recently, high-density single nucleotide polymorphism (SNP) arrays technology provides a superior approach to identify QTLs for wheat kernel-related traits [29–31]. To date, numerous QTLs for kernel traits have been identified on almost 21 wheat chromosomes [32–35]. Remarkably, major stable QTLs distributed on chromosomes 1A, 1B, 2D, 3D, 4A, 4B, 5A, 7D can be identified in recombinant inbred line (RIL) populations with different genetic backgrounds [36–40]. Moreover, several yield-related QTLs have been fine mapped and cloned, for example, the major QTL affecting kernel number and kernel weight on chromosome 2AL (*GNI-A1*) in tetraploid wheat [41, 42]. However, most QTLs associated with kernel traits were mapped by a low-density genetic linkage map with large confidence interval. Only a few QTLs flanking markers were converted into Kompetitive Allele Specific PCR (KASP) markers that can be used in molecular breeding.

Using a RIL population derived from ‘PuBing 3228 (P3228) × Gao8901 (G8901)’, the objectives of this study were to (i) identify stable and major QTLs for TKW, KL, KW and KL/W under different field conditions; (ii) reveal the contribution of the other kernel traits to TKW using conditional QTL analysis; (iii) predict candidate gene(s) for targeted QTLs interval based on reference genome annotation information; (iv) develop KASP markers of the candidate gene(s) and verified by PG-RIL and a natural population consisted of 141 cultivar/lines for marker-assisted selection in high-TKW wheat breeding.

## Results

### Phenotypic performance and correlation analysis

**Fig. 1:**
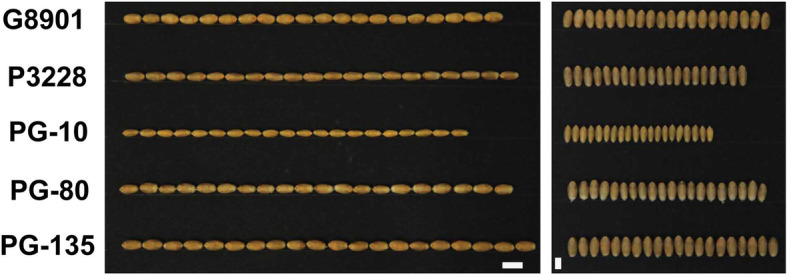
Phenotypic characterization of two parents and some representative RIL

**Fig. 2:**
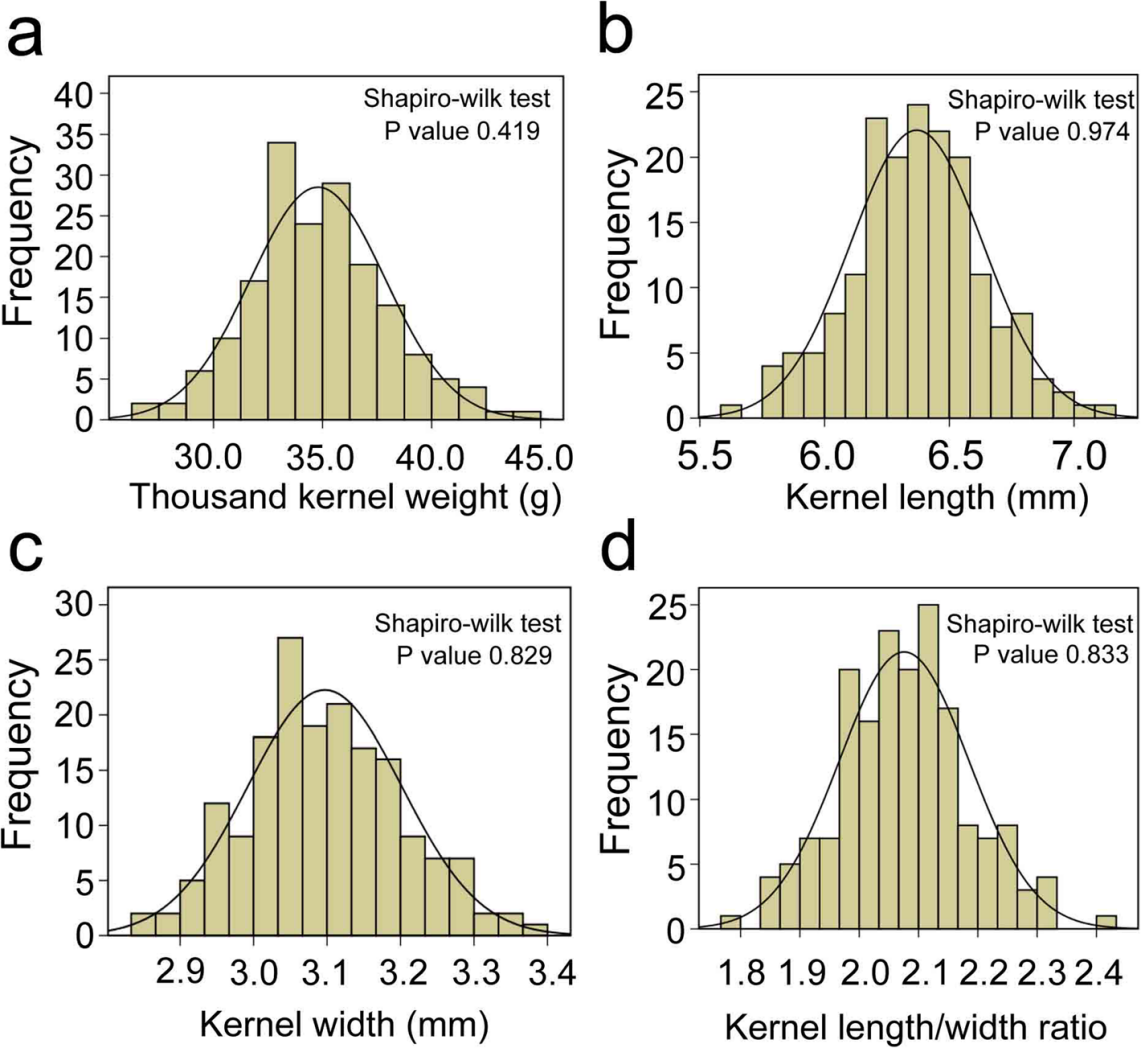
Frequency distribution of four kernel traits in RIL population in BLUP data. **a** Thousand kernel weight. **b** Kernel length. **c** Kernel width. **d** Kernel length/width

The 176 RIL population and their two parents P3228, G8901 were planted in four environments to identify stable and major QTLs for kernel-related traits. The means and ranges of four kernel-related traits (TKW, KL, KW and KL/W) are listed in Table 1. Compared with P3228, G8901 had wider KW, but shorter KL (Fig. 1 and Table 1). For the RIL population, the frequency of kernel traits in all environments and best linear unbiased predictors (BLUP) showed a continuous distribution with ranges from 27.33 to 44.97 g in TKW, 5.64 to 7.09 mm in KL, 2.84 to 3.39 mm in KW and 1.78 to 2.43 in KL/W (Table 1 and Fig. 2). The Shapiro-Wilk test and Pearson’s correlation coefficients of the four traits were calculated based on the BLUP data of four individual environments, indicating that TKW, KL, KW and KL/W showed normal distributions in multiple environments (Fig. 2 and Table 2). Moreover, TKW was positively correlated with KL and KW, and negatively correlated with KL/W (Table 2). The variance for genotype, environment and genotype × environment (GE) interaction effects were highly significant in TKW, KL, KW and KL/W (Additional file 1: Table S1). All the broad-sense heritability (*H*) of four traits were higher than 0.60 (Table 2), indicating that these traits were mainly determined by genetic factors.

**Table 1:** Phenotypes of the parents and PG-RIL population in this study

		Parents		PG-RILs					
Trait	Env	P3228	G8901	Min	Max	Mean	SD	CV(%)	*H*
TKW (g)	E1	31.68	36.93	20.79	46.77	32.54	4.37	13.42	0.668
	E2	38.23	44.22	24.18	45.86	35.05	3.63	10.35	
	E3	41.13	46.81	28.22	49.87	37.72	3.79	10.05	
	E4	33.64	40.95	23.43	46.32	33.82	4.12	12.18	
	BLUP	36.17	42.23	27.34	44.97	34.79	3.07	8.82	
KL (mm)	E1	6.70	6.36	5.53	7.22	6.33	0.31	4.85	0.859
	E2	6.67	6.29	5.50	7.04	6.25	0.29	4.61	
	E3	6.86	6.42	5.67	7.18	6.45	0.28	4.36	
	E4	6.78	6.46	5.62	7.21	6.45	0.29	4.45	
	BLUP	6.75	6.38	5.64	7.09	6.37	0.26	4.15	
KW (mm)	E1	2.91	3.23	2.52	3.32	2.95	0.16	5.32	0.615
	E2	3.02	3.29	2.64	3.34	3.03	0.13	4.32	
	E3	3.33	3.59	2.92	3.77	3.30	0.14	4.21	
	E4	3.08	3.42	2.73	3.52	3.10	0.15	4.77	
	BLUP	3.09	3.38	2.84	3.39	3.10	0.11	3.39	
KL/W	E1	2.30	1.97	1.83	2.61	2.16	0.14	6.48	0.796
	E2	2.21	1.91	1.76	2.46	2.07	0.12	5.80	
	E3	2.06	1.79	1.68	2.27	1.97	0.12	6.09	
	E4	2.20	1.89	1.80	2.52	2.10	0.12	5.71	
	BLUP	2.19	1.89	1.78	2.43	2.08	0.11	5.25	

Notes: TKW, thousand kernel weight; KL, kernel length; KW, kernel width; KL/W, kernel length/kernel width ratio; Env, environment; Min, minimum; Max, Maximum; BLUP, best linear unbiased predictors mean

**Table 2:** Correlation coefficients among the kernel traits of PG-RIL population in four environments

Trait	BLUP			E1			E2			E3			E4		
	TKW	KL	KW	TKW	KL	KW	TKW	KL	KW	TKW	KL	KW	TKW	KL	KW
KL	0.458^**^			0.387^**^			0.476^**^			0.432^**^			0.469^**^		
KW	0.823^**^	0.085		0.901^**^	0.133		0.792^**^	0.070		0.788^**^	0.0288		0.874^**^	0.194^**^	
KL/W	−0.235^**^	0.708^**^	− 0.641^**^	− 0.515^**^	0.536^**^	− 0.763^**^	− 0.204^**^	0.781^**^	− 0.566^**^	− 0.243^**^	0.707^**^	− 0.684^**^	− 0.356^**^	0.600^**^	− 0.665^**^

Note: ^*^ significant at *P* < 0.05 level; ^**^ significant at *P* < 0.01 level

### QTL mapping

**Fig. 3:**
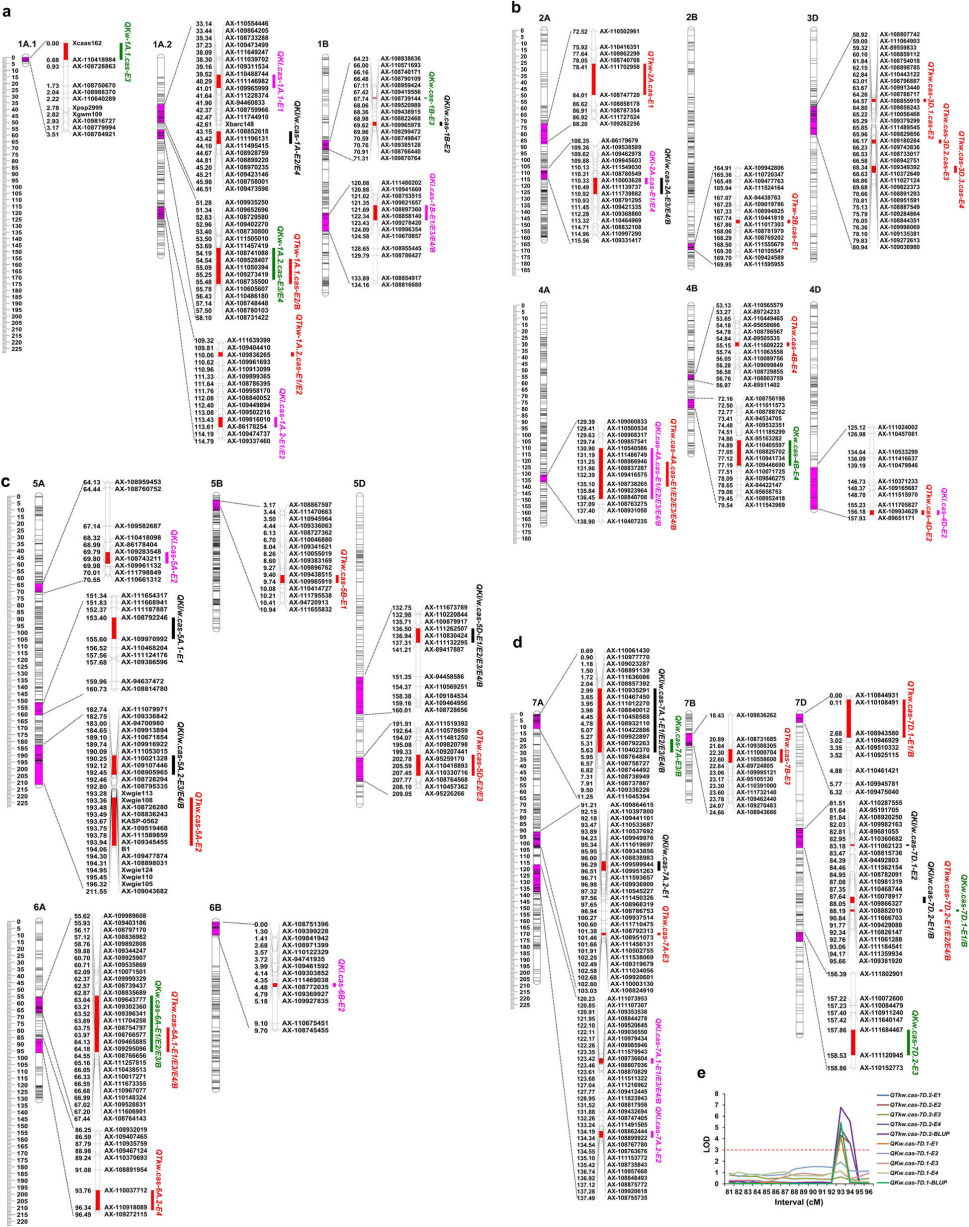
Genetic and physical locations of QTL regions associated with TKW, KL, KW and KL/W. **a** QTLs located on the chromosome 1A and 1B. **b** QTLs located on the chromosome 2A, 2B, 3D, 4A, 4B and 4D. **c** QTLs located on the chromosome 5A, 5B, 5D, 6A and 6B. (d) QTLs located on the chromosome 7A, 7B and 7D. (e) LOD curves for the QTLs *QTkw.cas-7D.2* and *QKw.cas-7D.1* on chromosome 7D. Uniform centimorgan (cM) scales are shown on the left. Physical maps are shown on the right of each genetic map. QTLs are indicated on the right side of each chromosome. For QTLs detected in different environments, a slash is inserted to distinguish the environments. The codes E1, E2, E3, E4 and B represent QTLs detected in 2013LC, 2014LC, 2015LC, 2016LC environments and BLUP data, respectively. Red, pink, green, black colors represent QTLs conferring TKW, KL, KW and KL/W, respectively

A total of 47 putative QTLs were detected for TKW, KL, KW and KW/L (Figs. 3a-3d and Additional file 1: Table S2). Among them, 25, eight and 13 QTLs were located on the A, B and D genome, respectively. The single QTL explained 1.79–22.41% of the phenotypic variance with threshold log-of-odds (LOD) value ranging from 2.54 to 11 (Additional file 1: Table S2). Seventeen stable QTLs could be detected in more than two individual environments (Fig. 3a-e and Table 3).

**Table 3:** Stable QTLs for thousand kernel weight, Kernel length, Kernel width, Kernel length/width traits in the PG-RIL population

Trait	QTL	Left Markers	Interval (cM)	E	PVE%	Add	Reference
TKW	*QTkw.cas-1A.2*	*AX-109528407* *–AX-108731422*	54.55–58.10	E2	5.93	−0.92	[8, 36, 43]
				E3	4.68	−0.84	
				E4	6.92	−1.13	
				BLUP	5.35	−0.74	
	*QTkw.cas-4A*	*AX-109416575* *–AX-108738265*	132.39–135.10	E1	10.59	−1.428	[40, 44]
				E2	8.31	−1.051	
				E3	11.84	−1.311	
				E4	10.69	−1.354	
				BLUP	13.17	−1.162	
	*QTkw.cas-5D*	*AX-109207441* *–AX-110418893*	199.33–205.59	E2	4.28	0.777	
				E3	3.28	0.699	
	*QTkw.cas-6A.1*	*AX-108835689* *–AX-110438513*	62.87–66.05	E1	6.52	1.137	[45, 46]
				E3	11.38	1.308	
				E4	12.73	1.526	
				BLUP	11.77	1.102	
	*QTkw.cas-7D.2*	*AX-111061288* *–AX-111184541*	92.76–93.06	E1	6.52	−1.150	[39, 43, 47]
				E2	5.50	−0.895	
				E4	6.09	−1.069	
				BLUP	6.22	−0.811	
KL	*QKl.cas-1A.2*	*AX-86178254* *–AX-109474737*	113.61–114.19	E1	4.29	0.061	
				E2	5.63	0.066	
	*QKl.cas-1B*	*AX-108897360* *–AX-110996354*	66.00–66.16	E1	6.63	0.075	[36]
				E3	8.16	0.073	
				E4	3.74	0.053	
				BLUP	7.38	0.071	
	*QKl.cas-2A*	*AX-108791295* *–AX-109421335*	110.93–111.45	E1	14.35	0.110	[37]
				E4	16.65	0.111	
	*QKl.cas-4A*	*AX-110540586* *–AX-108840708*	130.90–136.45	E1	7.48	−0.080	
				E2	8.46	−0.081	
				E3	8.09	−0.073	
				E4	9.66	−0.085	
				BLUP	9.00	−0.078	
	*QKl.cas-7A.1*	*AX-109353536* *–AX-109520645*	120.91–122.10	E1	5.78	−0.070	
				E3	7.24	−0.069	
				E4	5.70	−0.065	
				BLUP	7.24	−0.070	
KW	*QKw.cas-1A.2*	*AX-109402270* *–AX-108748448*	52.96–57.14	E3	5.55	−0.031	
				E4	10.14	−0.047	
	*QKw.cas-6A*	*AX-109892808* *–AX-110438513*	58.76–66.05	E1	5.43	0.037	[45, 48]
				E2	9.85	0.040	
				E3	7.31	0.036	
				BLUP	7.98	0.030	
KL/W	*QKl/w.cas-1A*	*AX-111196131* *–AX-108970235*	43.42–45.20	E2	22.41	0.093	
				E4	5.22	0.027	
	*QKl/w.cas-2A*	*AX-108791295* *–AX-109368860*	110.93–112.28	E3	11.60	0.040	
				E4	11.03	0.039	
				BLUP	10.90	0.037	
	*QKl/w.cas-5A.2*	*AX-94700980* *–AX-110671854*	183.00–189.10	E3	8.32	−0.034	
				E4	7.49	−0.032	
				BLUP	6.27	−0.028	
	*QKl/w.cas-5D*	*AX-110830424* *–AX-89417887*	136.94–141.21	E1	6.70	0.039	
				E2	3.77	0.038	
				E3	5.60	0.028	
				E4	5.58	0.028	
				BLUP	8.26	0.032	
	*QKl/w.cas-7A.1*	*AX-111636086* *–AX-109338226*	1.720–9.50	E1	13.84	0.055	
				E2	3.85	0.038	
				E3	12.25	0.041	
				E4	8.18	0.034	
				BLUP	13.23	0.041	

Notes: E: environments, BLUP: best linear unbiased predictors, PVE: phenotypic variance explained, Add: additive effect

A total of 19 QTLs for TKW were identified, of which 13 carried the favorable alleles from G8901 can increase the TKW, while the remaining six were from P3228 (Fig. 3a-d and Additional file 1: Table S2). In addition, five stable QTLs can be detected in at least two environments, including *QTkw.cas-1A.2*, *QTkw.cas-4A*, *QTkw.cas-5D*, *QTkw.cs-6A.1* and *QTkw.cas-7D.2* (Table 3). Remarkably, the major stable QTL *QTkw.cas-4A*, located on chromosome arm 4AL, can be repeatedly detected in all the environments and BLUP data, and phenotypic variance explained (PVE) ranged from 8.31 to 11.84% (Fig. 3b-c and Table 3). *QTkw.cas-6A.1* can be detected in the three environments as well as BLUP data, and the PVE ranged from 6.52 to 12.73% (Fig. 3c and Table 3). The favorable allele of *QTkw.cas-4A* was derived from the parent G8901, while *QTkw.cas-6A.1* was derived from the parent P3228. *QTkw.cas-1A.2*, *QTkw.cas-5D* and *QTkw.cas-7D.2* were three stable QTLs, with PVE at 4.68–5.93%, 3.28–4.28% and 5.50–6.52%, respectively (Table 3).

Ten QTLs for KL were detected, of which five QTLs (*QKl.cas-1A.2*, *QKl.cas-1B*, *QKl.cas-2A*, *QKl.cas-4A* and *QKl.cas-7A.1*) were significant in at least two environments (Figs. 3a-d, Table 3 and Additional file 1: Table S2). The major QTL *QKl.cas-2A* was significant in two environments, explaining 8.40–10.28% of the phenotypic variance (Fig. 3b and Table 3). Notably, the most stable QTL *QKl.cas-4A* was co-located with QTL *QTkw.cas-4A* for TKW (Fig. 3b and Table 3). Among the 10 QTLs for KL, six had additive effects from P3228 (Additional file 1: Table S2).

Eight QTLs for KW were identified on chromosomes 1A (two), 1B, 4B, 6A, 7A (two) and 7D, respectively (Figs. 3a-e, Table 3 and Additional file 1: Table S2). Among the three environments, the most stable QTL *QKw.cas-6A* in three environments was located on chromosome arm 6AS with PVE ranging from 5.43 to 9.85% (Fig. 3c and Table 3). This locus was co-located with the major QTL for TKW on 6AS (*QTkw.cas-6A.1*). The favorable alleles of the five QTLs (*QKw.cas-1A.2*, *QKw.cas-1B*, *QKw.cas-7A*, *QKw.cas-7D.1* and *QKw.cas-7D.2*) were derived from the parent G8901 (Figs. 3a-e, Table 3 and Additional file 1: Table S2).

A total of 10 QTLs for KL/W were identified on chromosomes 1A, 1B, 2A, 5A (two), 5D, 7A (two) and 7D (two), with PVE of individual QTL ranging from 1.79 to 22.41% (Figs. 3a-d, Table 3 and Additional file 1: Table S2). Five QTLs (*QKl/w.cas-1A*, *QKl/w.cas-2A*, *QKl/w.cas-5A.2*, *QKl/w.cas-7A.1* and *QKl/w.cas-7A.2)* were found in at least two environments (Table 3). Among them, the major stable QTL *QKl/w.cas-7A.1* can be detected in all the environments and BLUP data, explaining 3.85–13.84% of the phenotypic variance (Fig. 3d and Table 3). This QTL was co-located with QTLs for KW on chromosome 7A (*QKw.cas-7A*).

### Epistasis and QTL × environment interaction

A total of 15 pairs of epistasis QTLs for TKW, KL, KW and KW/L were detected, involving 30 QTLs on 15 chromosomes (Additional file 1: Table S3). Three pairs of epistasis interaction QTLs for TKW with PVE of 11.20, 7.10, and 8.93% were detected on chromosomes 1B/2D, 4D/6D, and 5A/6D, respectively, indicating that the interactions between those QTLs had no significant main effect on TKW (Additional file 1: Table S3). Three pairs of epistasis interaction sites of KL were detected, among which the interactions on chromosomes 4A/3B was between the major and non-major QTLs, while the interactions on 2D/3A and 6B/6D were between non-majors, and all of the three QTLs could increase KL (Additional file 1: Table S3). Four pairs of epistasis interactional QTLs for KW were detected, and they were all interactional between non-major QTLs. The two combinations of 3B/6A and 5B/6D could increase the KW, while the two combinations of 4B/6B and 5D/6B could decrease the KW. Five pairs of epistasis interactional QTLs for KL/W were detected, all of which were interactional between non-major QTLs. The two combinations of 6D/6D and 1B/6D could reduce KL/W, while the other three combinations could increase KL/W.

QTL × environment (QE) interactions were detected at 43 loci for TKW, KL, KW and KW/L (Additional file 1: Table S4). They overlapped with 47 putative QTLs of four traits, indicating that the TKW, KL, KW and KL/W were affected by environment. Among them, the largest environmental effect was detected in the interval *AX-109416575*–*AX-108738265* (PVE (AbyE) = 21.93%), indicating that the major QTLs *QTkw.cas-4A* and *QKl.cas-4A* for TKW and KL, respectively, were significantly affected by the environment (Additional file 1: Table S4). Ten pairs of epistasis interactions were detected for additive–additive–environment (AAE), including three, one, three and three pairs of epistasis QTLs for TKW, KL, KW and KL/W, respectively (Additional file 1: Table S3).

### QTL analysis for TKW conditioned on kernel-related traits

To dissect genetic effects of the KL, KW and KL/W on the expression of QTLs for TKW, conditional QTL analysis were conducted. After conditioned on KL, KW or KL/W, a total of 23 conditional QTLs comprising 47 QTL × environments were detected for TKW (Additional file 1: Table S5). Among them, 19 QTLs were identified as unconditional analysis, while the other 10 QTLs were newly detected, with four QTLs identified in at least two environments (Additional file 1: Table S5).

The QTLs *QTkw.cas-2A.1*, *QTkw.cas-4A* and *QTkw.cas-4D* were detected when TKW was conditioned on KW and KL/W instead of KL (Table 4 and Additional file 1: Table S5). This result indicated that these QTLs may be associated with KL, but independent of KW and KL/W. Four QTLs (*QTkw.cas-5A*, *QTkw.cas-6A.1*, *QTkw.cas-7A* and *QTkw.cas-7D.2*) were identified to be associated with KW, but independent of KL and KL/W (Table 4 and Additional file 1: Table S5). The QTL *QTkw.cas-1A.2*, was detected when TKW was conditioned on KL, but absent when conditioned on KW or KL/W (Table 4), suggesting that it may be independent of KL, but was associated with either one or both of KW and KL/W. The stable QTL *QTkw.cas-5D* was not detected when TKW was conditioned on KL, KW or KL/W (Table 4).

**Table 4:** Unconditional and conditional stable QTLs for TKW in wheat

QTL	Intervals marker	Unconditional QTL			Conditional QTL								
		TKW			TKW|KL			TKW|KW			TKW/(KL/W)		
		E	PVE%	Add	E	PVE%	Add	E	PVE%	Add	E	PVE%	Add
*QTkw.cas-1A.2*	*AX-109528407*–*AX-108731422*	E2	5.927	−0.915									
		E3	4.684	−0.836									
		E4	6.924	−1.127	E4	5.695	−0.893 b						
*QTkw.cas-4A*	*AX-109416575*–*AX-108738265*	E1	10.586	−1.428				E1	9.033	−0.654 b	E1	8.936	−1.372 a
		E2	8.310	−1.051							E2	7.278	−0.999 a
		E3	11.837	−1.311				E3	6.509	−0.562b	E3	11.238	−1.191 a
		E4	10.687	−1.354				E4	9.763	−0.687 b	E4	12.060	−1.301 a
*QTkw.cas-5D*	*AX-109207441*–*AX-110418893*	E2	4.280	0.777									
		E3	3.280	0.699									
*QTkw.cas-6A.1*	*AX-108835689*–*AX-110438513*	E1	6.515	1.137	E1	5.812	0.989 b				E2	6.208	0.832 b
		E3	11.380	1.308	E3	11.609	1.257 a						
		E4	12.727	1.526	E4	11.069	1.244 b				E4	9.719	1.171 b
*QTkw.cas-7D.2*	*AX-111061288*–*AX-111184541*	E1	6.520	−1.150	E1	11.086	−1.381 c						
		E2	5.502	−0.895	E2	7.700	−1.014 c						
					E3	4.806	−0.819 d						
		E4	6.091	−1.069	E4	6.946	−0.997 a				E4	4.649	−0.820 b

Note: ^a^denotes the additive effect of a conditional QTL, in absolute values, that reduces or increase less than 10% compared to the corresponding unconditional QTL

^b^denotes the additive effect of a conditional QTL, in absolute values, that reduces more than 10% compared to the corresponding unconditional QTL

^c^denotes the additive effect of a conditional QTL, in absolute values, that increase more than 10% compared to the corresponding unconditional QTL. ^d^denotes the QTL couldn’t be detected in unconditional analysis, but can be detected in conditional analysis

(+) indicates that the most favorable allele is derived from the parent P3228, (−) indicates that the most favorable allele is derived from the parent G8901. E and numerals in parentheses indicate the environment in which the QTL was detected and the percentage of phenotypic variance explained (PVE) by the additive effects of the mapped QTLs, respectively

### Important QTL clusters

A total of seven QTL clusters were identified, all of them were related to more than one trait (Fig. 3a-d and Table 5). Three intervals harboring various QTLs can be identified in at least three environments (Fig. 3a-d, Tables 3 and 5). The interval *AX-110540586*–*AX*-*108840708* on chromosome 4A affected TKW and KL across all the four environments and BLUP data, and the additional effects were derived from G8901 (Fig. 3a-d, Tables 3 and 5). The interval *AX-109892808*–*AX-110438513* on chromosome 6A affected TKW and KW across the three environments and BLUP data, with P3228 conferring the favorite allele (Fig. 3c and Table 5). The interval *AX-111061288*–*AX-111184541* on chromosome 7D showed significant effects on TKW and KW across three environments and BLUP data and on KL/W in one environment and BLUP data (Table 5 and Fig. 3d). In this interval, the G8901-derived allele increased TKW and KW and decreased KL/W (Table 3).

**Table 5:** Characterization of QTL clusters for kernel traits in this study

Clusters	Chromosomes	Intervals marker	Intervals (cM)	QTL included	No of QTLs	Traits (additive effect, number of environments)^a^
C1	1A	*AX-111196131–AX-108731422*	43.42–58.07	*QTkw.cas-1A.2*, *QKw.cas-1A.2*, ***QKl/w.cas-1A***	3	TKW(−3), KW(−2), **KL/W(+ 2)**
C2	2A	*AX-108791295*–*AX-109368860*	110.93–112.28	***QKl.cas-2A***, ***QKl/w.cas-2A***	2	**KL(+ 2)**, **KL/W(+ 2)**
C3	4A	*AX-110540586*–*AX-108840708*	130.91–136.45	***QTkw.cas-4A***, *QKl.cas-4A*	2	**TKW(−4)**, KL(−4)
C4	4D	*AX-109934629*–*AX-89651171*	156.18–157.93	*QTkw.cas-4D*, *QKl.cas-4D*	2	TKW(+ 1), KL(+ 1)
C5	6A	*AX-109892808*–*AX-110438513*	58.76–66.05	***QTkw.cas-6A.1***, *QKw.cas-6A*	2	**TKW(+ 3)**, KW(+ 3)
C6	7A	*AX-111636086*–*AX-109338226*	1.72–9.50	***QKw.cas-7A***, ***QKl/w.cas-7A.1***	2	**KW(−1)**, **KL/W(+ 4)**
C7	7D	*AX-111666703*–*AX-111184541*	90.84–93.06	*QTkw.cas-7D.2*, *QKw.cas-7D.1*, *QKl/w.cas-7D.2*	3	TKW(−3), KW(−1), KL/W(+ 1)

Notes: ^a^A trait name in bold type indicates that major QTLs were detected for the corresponding trait, and a trait name in underlined type indicates that stable QTLs were detected for the corresponding traits. (+) indicates that the most favorable allele is derived from the parent P3228, (−) indicates that the most favorable allele is derived from the parent G8901

### Predicting of candidate gene *TaFT-D1* for QTLs *QTkw.cas-7D.2* and *QKw.cas -7D.1*

The two stable QTLs, *QTkw.cas-7D.2* and *QKw.cas-7D.1*, was delimited by the markers *AX-110826147* and *AX-111359934* (Fig. 3d), and the peak interval were co-located between the markers *AX-111061288* and *AX-111184541* (Table 3 and Fig. 3d-e). Collinearity analysis indicated that the genetic map of PG-RIL and the physical map of Chinese Spring reference genome V1.0 show perfect collinearity in the chromosomes 7DS region (Additional file 2: Fig. S1). To investigate the physical intervals of QTLs *QTkw.cas-7D.2* and *QKw.cas-7D.1*, we aligned the markers *AX-110826147* and *AX-111359934* to Chinese Spring reference genome V1.0 [49]. The results showed that the physical interval of QTLs *QTkw.cas-7D.2* and *QKw.cas-7D.1* is mapped to the 65.50–69.32 Mb position on chromosome arm 7DS which contained 47 high confidence genes (Table 3 and Additional file 2: Table. S2).

Subsequently, we annotated 47 genes in the 3.82 Mb region (Additional file 2: Table. S3). Among them, *TaFT-D1* (*TraesCS7D02G111600*), a homolog of Arabidopsis *FLOWERING LOCUS T*, was considered as the candidate gene for *QTkw.cas-7D.2* and *QKw.cas-7D.1* (Additional file 1: Tables S6). Then, we designed genome-specific primers for sequencing to analyse the genome sequence of *TaFT-D1* from G8901 and P3228 (Additional file 1: Tables S9), and found that there was a 1 bp deletion at position + 840 in the third exon of *TaFT-D1* in P3228. Protein sequence alignment revealed that this deletion caused frameshift mutation with loss function of the TaFT-D1 protein in P3228 (Additional file 2: Fig. S2). We further analyzed the expression profiles of 47 candidate genes in different tissues using the Chinese Spring cv-1 development (pair) database [50]. As shown in Additional file 2: Fig. S3, the expression of *TaFT-D1* was highest in leaves and young spikes, slightly lower in stems and substantially lower in root and developing grain.

### Development of KASP markers and analysis for alleles of *TaFT-D1*

**Fig. 4:**
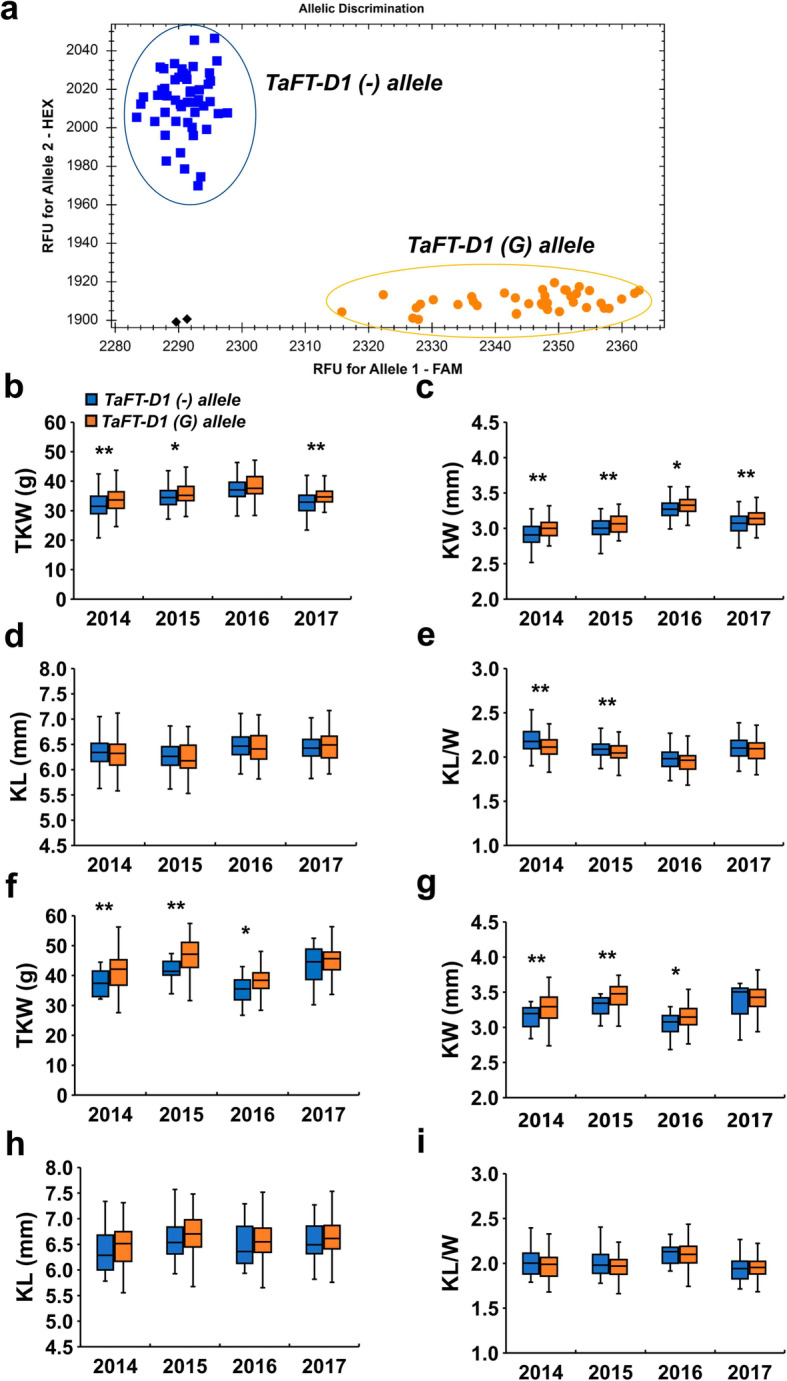
Allelic analysis with kernel traits of *TaFT-D1* in PG-RIL and the natural population. **a** Allelic segregation of KASP marker for *TaFT-D1 alleles*. Comparison analysis of *TaFT-D1* alleles with the thousand kernel weight (TKW, **b**), kernel length (KL, **c**), kernel width (KW, **d**) and kernel length/width (KL/W, **e**) of PG-RIL in four environments. Comparison analysis of *TaFT-D1* alleles with the TKW (**f**), KL (**g**), KW (**h**) and KL/W (**i**) of the natural population consisted of 141 cultivar/lines in four environments. ^**^P < 0.01 and ^*^P < 0.05 (two-tailed t test) indicates a significant difference to the two haplotypes

Two SNPs markers (*AX-111061288* and *AX-111184541*) closely linked to the two stable QTLs (*QTkw.cas-7D.2* and *QKw.cas-7D.1*) and 1 bp InDel of *TaFT-D1* were further converted to KASP markers (Fig. 4a, Additional file 2: Fig. S4 and Additional file 1: Tables S9). After screening PG-RIL and a natural population consisted of 141 cultivar/lines using these KASP markers, we found that the KASP marker of *TaFT-D1* was co-segregated with SNPs marker *AX-111184541*. This result further proved that *TaFT-D1* was an important candidate gene for the *QTkw.cas-7D.2* and *QKw.cas-7D.1*. Furthermore, two-tailed t test was performed between the InDel of *TaFT-D1* and four kernel-related traits collected from multiple environments. The results showed that the InDel of *TaFT-D1* was significantly correlated with TKW, KW and KL/W but not with KL for PG-RIL (Fig. 4b-e). For the natural population consisted of 141 cultivar/lines, the InDel of *TaFT-D1* was associated with TKW and KW in the three environments, except that no significant differences were observed in the KL and KL/W of G8901-allele (*TaFT-D1(G)-allele*) and P3228-allele (*TaFT-D1(−)-allele*) plants (Figs. 4f-i). The mean TKW of *TaFT-D1(G)-allele* was significantly higher than those of the *TaFT-D1(−)-allele* (mean 4.91 g higher in 2013–2014, 5.21 g higher in 2014–2015, 2.87 g higher in 2015–2016 and 1.58 g higher in 2016–2017).

### *TaFT-D1(G)-allele* underwent positive selection during Chinese wheat breeding

**Fig. 5:**
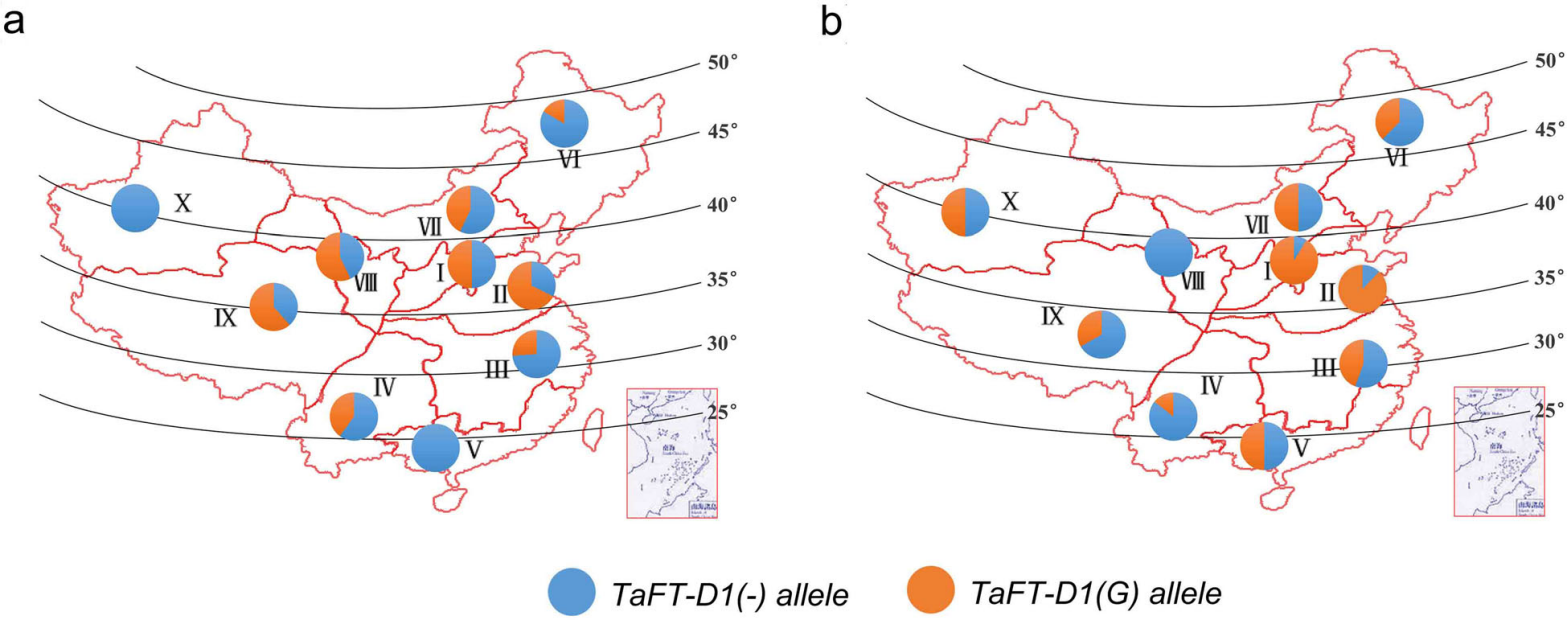
Geographic distribution of the *TaFT-D1* alleles in the Chinese wheat ecological regions. Distribution of *TaFT-D1* alleles in landraces (**a**) and modern cultivars (**b**) among ten Chinese ecological regions. I, northern winter wheat region; II, Yellow and Huai River valley winter wheat region; III, low and middle Yangtze River valley winter wheat region; IV, southwestern winter wheat region; V, southern winter wheat region; VI, northeastern spring wheat region; VII, northern spring wheat region; VIII, northwestern spring wheat region; IX, Qinghai–Tibet spring–winter wheat region; X, Xinjiang winter–spring wheat region

To determine whether the two *TaFT-D1* alleles were subjected to selecting, we investigated the geographic distribution of the *TaFT-D1* alleles in 150 Chinese wheat landraces and 172 modern cultivars. The Chinese wheat production area is divided into 10 agro-ecological wheat production regions according to environment, type of cultivars and growing season [51, 52]. Compared with landraces, the proportion of *TaFT-D1(G)-allele* in modern cultivars was higher in the seven agro-ecological wheat production regions (except for regions IV, VIII and IX), suggesting that *TaFT-D1(G)-allele* have undergone positive selection during wheat breeding process (Fig. 5a and b). This confirmed that the favorable *TaFT-D1(G)-allele* can be used in different wheat production regions.

## Discussion

### Unconditional QTLs and conditional QTLs effects

Previous researches have shown that the combination of QTL mapping and conditional genetic analysis enable the identification of the influence of one trait on another [22, 28]. In the current study, we dissected QTLs based on TKW values conditioned on KL, KW and KL/W to study the genetic basis of TKW on QTL expression. When conditioned on KW, four conditional stable QTLs (*QTkw.cas-1A.2*, *QTkw.cas-5D*, *QTkw.cas-6A.1*, *QTkw.cas-7D.2*) account for TKW, while two (*QTkw.cas-4A* and *QTkw.cas-5D*) on KL (Table 4). Notably, *QTkw.cas-5D* was not detected when TKW was conditioned on KL or KW (Table 4). The total PVE of the four QTLs conditioned on KW was significantly higher than the two on KL, indicating that KW contributes more than KL to TKW in the PG-RIL population (Table 4). The unconditional QTL analysis showed that the major QTL *QTkw.cas-4A* on chromosome 4A was co-located with QTL *QKl.cas-4A* for KL, with G8901-derived allele increasing both TKW and KL (Table 3 and Fig. 3b). Using conditional QTL analysis, we found that the *QTkw.cas-4A* was entirely contributed by KL, partially by KW and entirely independent by KL/W (Table 4). Combining unconditional QTL with conditional QTLs analysis, the effect of increasing TKW of *QTkw.cas-4A* was identified to be mainly caused by the KL. Using the same analysis methods, we concluded that the effects of increasing TKW of *QTkw.cas-6A* and *QTkw.cas-7D* were mainly contributed by the KW. The results should be valuable for dissecting the genetic basis of TKW and the genetic contribution of kernel related traits to TKW at individual QTL level in wheat.

### QTL comparison

To date, a large number of QTLs for TKW and kernel morphological traits have been mapped in common wheat [45, 48]. To investigate whether there were overlapping QTLs in different genetic backgrounds, we compared the QTLs interval in this study with those in the previous studies. Some stable QTLs have been reported in the previous studies. For example, the interval *AX-108835689*–*AX-110438513* on chromosome 6A contained *QTkw.cas-6A.1* and *QKw.cas-6A*, corresponding to the reported QTLs for kernel weight in different RIL population [44–46]. The gene *TaGW2-A1* was also located in this interval*,* and it affects TKW by regulating the KW of bread wheat [13, 52]. It was also reported that the major stable QTLs *QTkw.cas-4A* and *QKl.cas-4A* were in the interval *AX-108738265*–*AX-109416575* (Table 5), overlapping with the locus for TKW in the previous study [40, 47]. The QTL *QTkw.cas-7D* in the interval *AX-111061288*–*AX-111184541* on chromosome 7D has also reported previously [39, 43, 53, 54]. Therefore, these important QTLs that were not affected by genetic background are important selection targets in wheat breeding.

### Advantages of high-density genetic maps

Previous genetic maps were mainly constructed by gel-based markers. Moreover, the confidence intervals associated with detected QTLs were relatively large and the numbers of markers was limited, which restricted further fine mapping of QTLs and their applications in breeding [27, 38]. Compared with gel-based markers, high-density SNP arrays have the advantage of abundant markers and can further reduce the confidence interval for QTL localization. In this study, we used the wheat 660 K high-density SNP chips to screen the PG-RIL population, and found that the confidence interval for most QTLs was less than 3 cM (Table 3 and Additional file 1: Table S2). Furthermore, the SNP markers in the confidence interval have clear base sequence and position information, which is effective for fine mapping using the reference genome [27]. For instance, the stable QTL *QTkw.cas-7D.2* and *QKw.cas-7D.1* were co-located in interval between 92.756–93.059 cM, and the physical interval of the Chinese Spring reference genome V1.0 is 65.50–69.32 Mb (Table 3 and Fig. 3).

### Functional prediction of candidate genes for *QTkw.cas-7D.2* and *QKw.cas-7D.1*

In crops, genes that regulated flowering have diverse functions, some affecting the yield-related traits [54]. Kernel weight can be manipulated by altering the duration of kernel filling, which is greatly influenced by flowering-related genes. For instance, overexpression of *TaGW8*, the positive regulator of cell proliferation and grain filling, results in early flowering and enhanced kernel width and yield in wheat [55, 56]. Overexpression of *TaZIM-A1* represses the expression of *TaFT1*, leading to a delay in heading date and decreased TKW in common wheat [57]. In the present study, the stable QTLs *QTkw.cas-7D.2* and *QKw.cas-7D.1* were delimited to the 3.82 Mb physical interval with 47 high-confidence genes (Additional file 1: Table S6). Among them, compared with G8901, frameshift mutation of *TaFT-D1* in P3228 leads to loss of protein function (Additional file 2: Fig. S2). *TaFT1*, a homolog gene of Arabidopsis *FLOWERING LOCUS T*, is a major gene that regulates wheat flowering [58, 59]. It has diverse functions on regulating different reproductive traits, such as flowering time, spike development and seed development [60, 61]. The loss function of *TaFT-D1* in P3228-allele lines resulted in delayed flowering and decreased TKW, while the high expression of *TaFT-D1* in the G8901-allele lines leads to accelerated flowering time and increased TKW.

### Diagnostic marker and marker-assisted selection

Abundance of diagnostic markers in wheat enables breeders to create better combinations and select favorable cultivars to meet local breeding goals [62]. To date, numerous SNP loci related to kernel traits have been identified in wheat by high-throughput SNP chips combined with bi-parental populations [1, 34, 39]. In the present study, a KASP marker was developed to distinguish two alleles of *TaFT-D1* and verified in PG-RIL and a natural population consisted of 141 cultivar/lines (Fig. 4). Furthermore, the alleles of *TaFT-D1* were significantly associated with TKW and KW in both PG-RIL and natural populations (Fig. 4). G8901-allele, the favorable allele that produces higher TKW, was gradually accumulated during the wheat breeding process (Fig. 5). Therefore, the KASP marker can facilitate map-based cloning of *QTkw.cas-7D.2* and *QKw.cas-7D.1* and molecular-assisted selection breeding for high-yield in wheat.

## Conclusions

In this study, we performed QTL analysis using the PG-RIL population in four environments for kernel-related traits (TKW, KL, KW and KL/W), which were mainly distributed on chromosomes 1A, 1B, 4A, 5D, 6A, 7A and 7D (Fig. 3 and Additional file 2: Table S1). A total of 17 stable QTLs were identified in more than two individual environments (Table 3). Notably, the stable QTLs for TKW were mainly affected by KW (Table 4). Furthermore, the QTLs *QTkw.cas-7D.2* and *QKw.cas-7D.1* were delimited to the physical interval of approximately 3.82 Mb, and *TaFT-D1* was considered as the candidate gene. Based on a 1 bp InDel of *TaFT-D1* between the two parents, a KASP marker of *TaFT-D1* allele was developed and verified by PG-RIL and a natural population. The favorable *TaFT-D1 (G)-allele* associated with TKW and KW has been positively selected during Chinese wheat breeding. In addition, the current study provided new options for dissecting the genetic basis of yield and molecular-assisted breeding.

## Methods

### Plant materials and field trials

A mapping population composed of 176 F_6–9_ RILs derived from ‘PuBing3228 × Gao 8901 ‘was developed by single seed descent method. The wheat germplasm P3228 was developed by Dr. Lihui Li (Chinese Academy of Agricultural Sciences). G8901 is a commercial cultivar released by Gaocheng institute of agricultural science, Hebei, China. The P3228 has higher kernel number per spike and the G8901 has higher thousand kernel weight (Fig. 1). A natural population consisted of 141 cultivar/lines (maintained in our laboratory, Additional file 1: Table S7) was used for the KASP marker screening and two-tailed t test. The 176 RILs, with two parents and the natural population were grown at the Luancheng Agro-ecosystem Station, Chinese Academy of Sciences (37°53′15″N, 114°40′47″E) during four growing seasons from 2013 to 2014 to 2016–2017. In each environment, the RILs, two parents and the natural population were planted in a randomized complete block design with three replicates. A 1.5 m^2^ subplot with four 1.5 m-long rows, 0.25 m apart, and 30 seeds for each row were used. The water, fertilizer and other management of all field trials were carried out in accordance with local standard practices. In addition, 150 landraces of the Chinese wheat mini-core collection [63] and 172 modern cultivars (maintained in our laboratory) were used to analyze the geographic distribution of *TaFT-D1* alleles (Additional file 1: Table S8). The 150 landraces and 88 modern cultivars of the Chinese wheat mini-core collection were kindly provided by Dr. Xueyong Zhang (Chinese Academy of Agricultural Sciences).

### Phenotypic evaluation and statistical analysis

For the four environments, 10 representative plants were sampled from each plot to investigate kernel-related traits. At seed maturity, the TKW and kernel morphometric traits (KL, KW and KL/W) of at least 500 kernels were measured three times using the rapid SC-G grain appearance quality image analysis system (WSeen Detection, Hangzhou, China). Analysis of variance (ANOVA), mean values of traits, standard deviations and variation coefficients (CV) were performed with SPSS Statistics v20.0 software (SPSS, Chicago, USA). Effects among genotypes, environments, and GE interaction were estimated by ANOVA. BLUP for all four traits across four environments was calculated using R software (V.3.2.2; https://www.r-project.org/). The *H* was calculated using the QGAStation 2.0 (http://ibi.zju.edu.cn/software/qga/v2.0/index c.htm) and the following formula *H* = *V*G/*V*P; where *V*G and *V*P are the genetic variance and phenotypic variance, respectively.

### QTL mapping

The ‘PuBing3228 × Gao 8901’ RIL population and the two parents were genotyped by the Affymetrix wheat 660 K SNP array [64]. A total of 101,136 loci showed polymorphisms between P3228 and G8901. The linkage map comprised 23 linkage groups that consisted of 4477 bins, spanning 3529.5 cM in length, with an average interval distance of 0.782 cM between the adjacent markers. Linkage analysis was performed using JoinMap v4 [65], and the genetic map was drawn by Mapchart 2.0 [66]. The QTLs were scanned with QTL ICIMapping V4.1 [67] through inclusive composite interval mapping of additive and dominant QTL (ICIM-ADD) [67]. The LOD score to detect the presence of a QTL was above at 2.50 [68]. Digenic epistasis and environment interaction of QTLs were analyzed using QTL ICIMapping V4.1 through inclusive composite interval mapping of epistatic QTL (ICIM-EPI) [69]. The LOD score to detect the digenic epistasis QTL was above at 5.0 [68]. The QTL × environment interactions were scanned with QTL ICIMapping V4.1 through inclusive composite interval mapping of additive and dominant QTL (ICIM-ADD) [70]. QTLs with overlapping confidence intervals were regarded as the congruent QTLs. The QTLs were named based on McIntosh et al. [71], ‘cas’ represents Chinese Academy of Science.

Conditional QTL analysis was performed to analyze the genetic contributions of kernel-related traits to TKW, by the procedure of inclusive composite interval mapping [23]. The conditional phenotypic values (*y*_(TKW|KL)_) of TKW in wheat were obtained by the mixed-model approach. The conditional phenotypic value can be partitioned as.

*y*_(TKW|KL)=_μ_(TKW|KL)_ + *G*_(TKW|KL)_ + *E*_(TKW|KL)_ + *e*_(TKW|KL)_.

where (TKW|KL) denote TKW conditional on KL; *y*_(TKW|KL)_ is the conditional phenotypic value of TKW on KL; μ_(TKW|KL)_ is the conditional population mean, *G*_(TKW|KL)_ is the conditional general genotypic effect; *E*_(TKW|KL)_ is the conditional effect for the environment and *e*_(TKW|KL)_ is the conditional residual error.

The conditional phenotypic values (*y*_(TKW|KL)_, *y*_(TKW|KW)_ and *y*_(TKW|KL/W)_) are the conditional phenotypic value of TKW on KL, KW or KL/KW in the corresponding environment, which were estimated using QGAStation2.0 (http://ibi.zju.edu.cn/software/qga/). MapChart 2.2 (http://www.biometris.nl/uk/Software/MapChart/) was used to draw the genetic map.

### Comparison of QTLs related to kernel traits

We used flanking SNP markers sequence of QTLs to BLAST against the reference genome of Chinese Spring to acquire the physical position of the region [49]. High confidence candidate genes in the target interval were retrieved based on coding sequences (IWGSC_RefSeq_Annotations_v1.0), and were further analyzed on NCBI Non-redundant protein sequences for function annotations. The expression profile database of nine candidate genes was blasted based on Chinese Spring cv-1 Development (pair) [50].

### Conversion of SNPs to KASP markers

The SNPs tightly linked to two stable QTLs *QTkw.cas-7D.2* and *QKw.cas-7D.1* and the 1 bp InDel of *TaFT-D1* were converted to KASP markers (Additional file 1: Table S9). KASP reactions were carried out on a BIORAD CFX real-time PCR system using the KASP V4.0 2× Mastermix (LGC Genomics, Teddington, UK) as previously described [1]. The fluorescence was monitored using Bio-Rad CFX Manage 3.1 software (LGC Genomics, Teddington, UK). Two-tailed t test was performed with SPSS Statistics v20.0 software (SPSS, Chicago, USA).

## Supplementary information

**Additional file 1 Table S1** Analysis of variance for the investigated traits of the PG-RIL in four environments. **Table S2** Putative additive QTL associated with kernel related traits in four environments. **Table S3** Epistatic effects and environmental interactions of QTLs for TKW, KL, KW and KL/W in wheat detected from the PG-RIL population. **Table S4** QTL × environment interactions for TKW, KL, KW and KL/W in wheat detected from the PG-RIL population. **Table S5** Unconditional and conditional QTLs for TKW in wheat. **Table S6** Annotated genes harbored in the interval of *QTkw.cas-7D.2* and *QKw.cas-7D.1*. **Table S7** Detailed information on a natural population consisted of 141 cultivar/lines and their alleles of *TaFT-D1*. **Table S8** Detailed information on 150 landraces and 172 modern cultivars and their alleles of *TaFT-D1*. **Table S9** Primers used in this study.

**Additional file 2 Fig. S1** Collinearity between the genetic (left) and physical (right) positions for SNPs mapped on the chromosome 7DS in PG-RIL genetic map. **Fig. S2** A 1 bp InDel in *TaFT-D1* caused a frameshift mutation of the protein. (a) Sequence alignment of *TaFT-D1* showing 1 bp InDel between P3228 and G8901. (b) Protein alignment of TaFT-D1 showing frameshift mutation in P3228. **Fig. S3** Heatmap showing the expression profile of DEGs at 15 development stages. **Fig. S4** Allelic segregation of KASP markers *AX-111061288* (a) and *AX-111184541* (b) for *QTkw.cas-7D.2* and *QKw.cas-7D.1*.

## Data Availability

All the data generated or analyzed during the current study were included in the manuscript and its additional files. The raw data is available from the corresponding author on reasonable request. The collection of materials used in current study complied with institutional, national, or international guidelines.

## Abbreviations

BLUP: best linear unbiased predictors
ICIM: inclusive composite interval mapping
G8901: Gao8901
KASP: Kompetitive Allele-Specific PCR
KL: kernel length
KW: kernel width
LOD: threshold log-of-odds
P3228: PuBing 3228
PG-RIL: ‘PuBing3228 × Gao 8901’ recombinant inbred lines
PVE: phenotypic variance explained
QTL: quantitative trait loci
SNP: single nucleotide polymorphism
TKW: thousand kernel weight
