# Supplementary material for: Identification and validation of quantitative trait loci for kernel traits in common wheat (Triticum aestivum L.)
Source: BMC Plant Biol. 2020 Nov 23;20:529. doi: 10.1186/s12870-020-02661-4 (PMC7682089; doi:10.1186/s12870-020-02661-4)
Supplement: Supplementary file 2 — Additional file 2 Fig. S1 Collinearity between the genetic (left) and physical (right) positions for SNPs mapped on the chromosome 7DS in PG-RIL genetic map. Fig. S2 A 1 bp InDel in TaFT-D1 caused a frameshift mutation of the protein. (a) Sequence alignment of TaFT-D1 showing 1 bp InDel between P3228 and G8901. (b) Protein alignment of TaFT-D1 showing frameshift mutation in P3228. Fig. S3 Heatmap showing the expression profile of DEGs at 15 development stages. Fig. S4 Allelic segregation of KASP markers AX-111061288 (a) and AX-111184541 (b) for QTkw.cas-7D.2 and QKw.cas-7D.1. [file 12870_2020_2661_MOESM2_ESM.pdf]

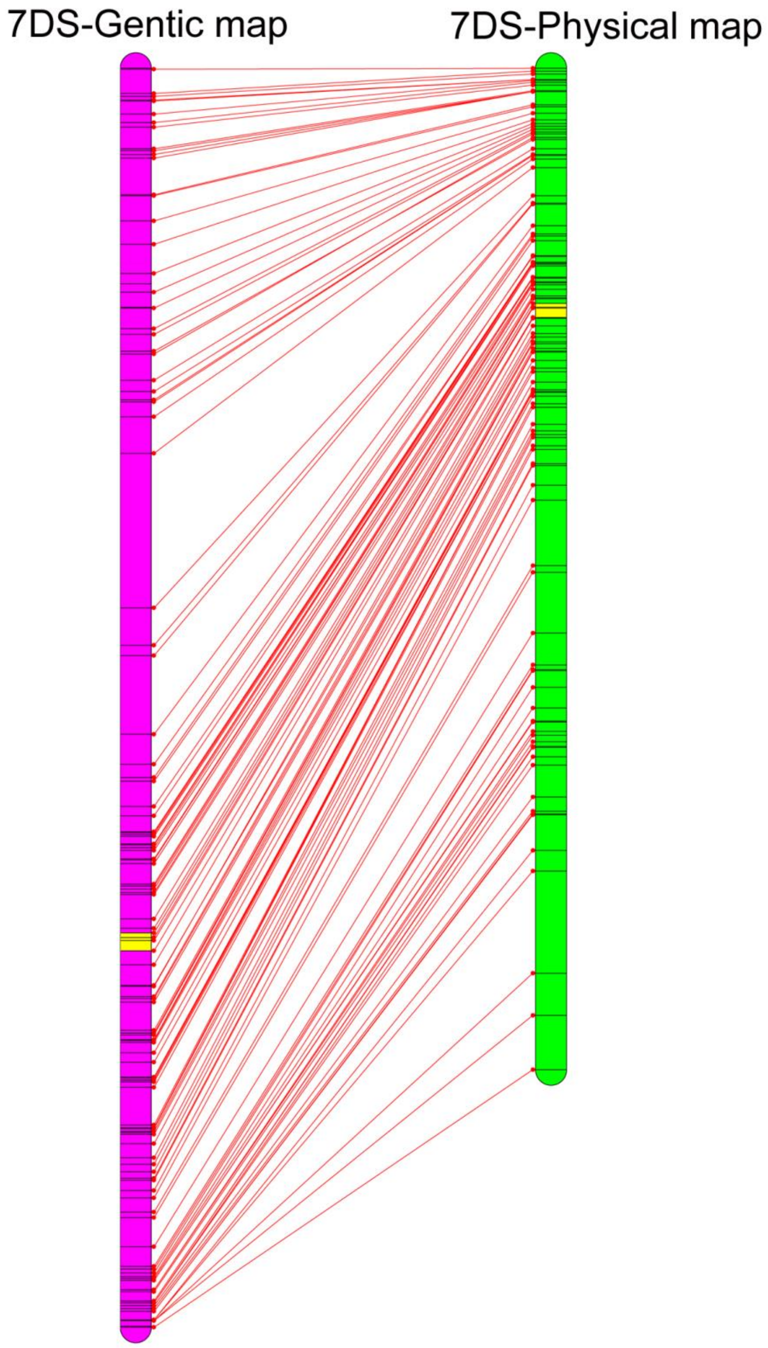

Supplementary Fig. S1 Collinearity between the genetic (left) and physical (right) positions for SNPs mapped on the chromosome 7DS in PG-RIL genetic map

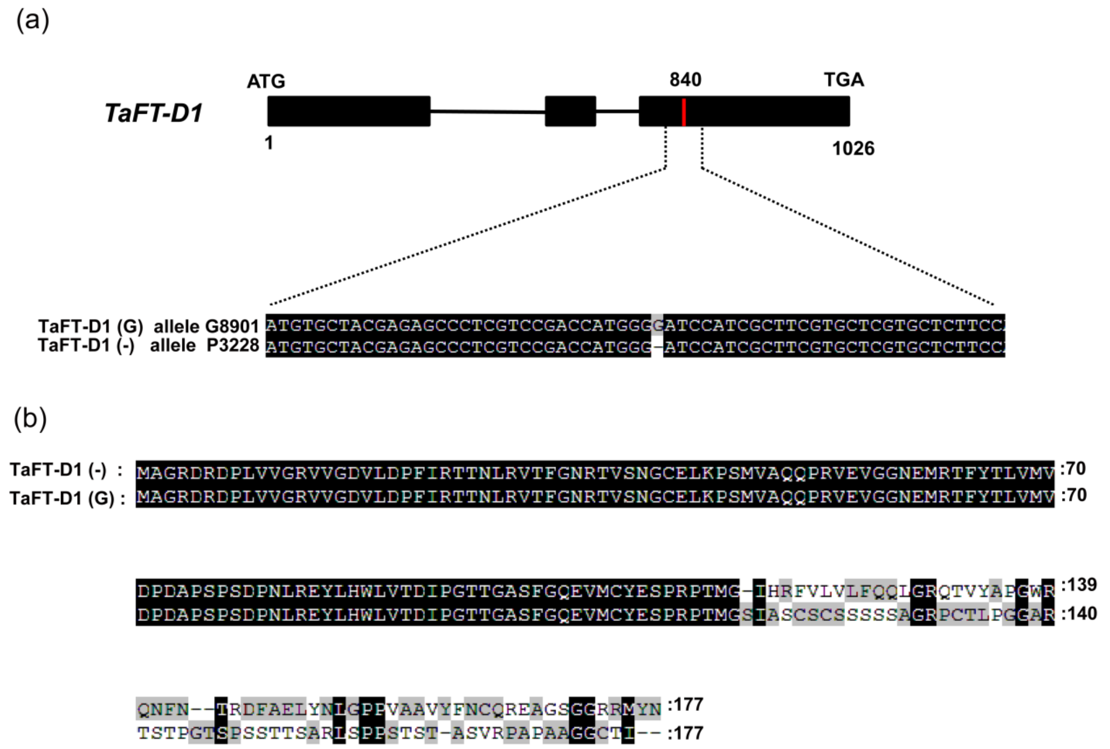

Supplementary Fig. S2 A 1bp InDel in *TaFT-D1* caused a frameshift mutation of the protein. (a) Sequence alignment of *TaFT-D1* showing 1 bp InDel between P3228 and G8901. (b) Protein alignment of TaFT-D1 showing frameshift mutation in P3228.

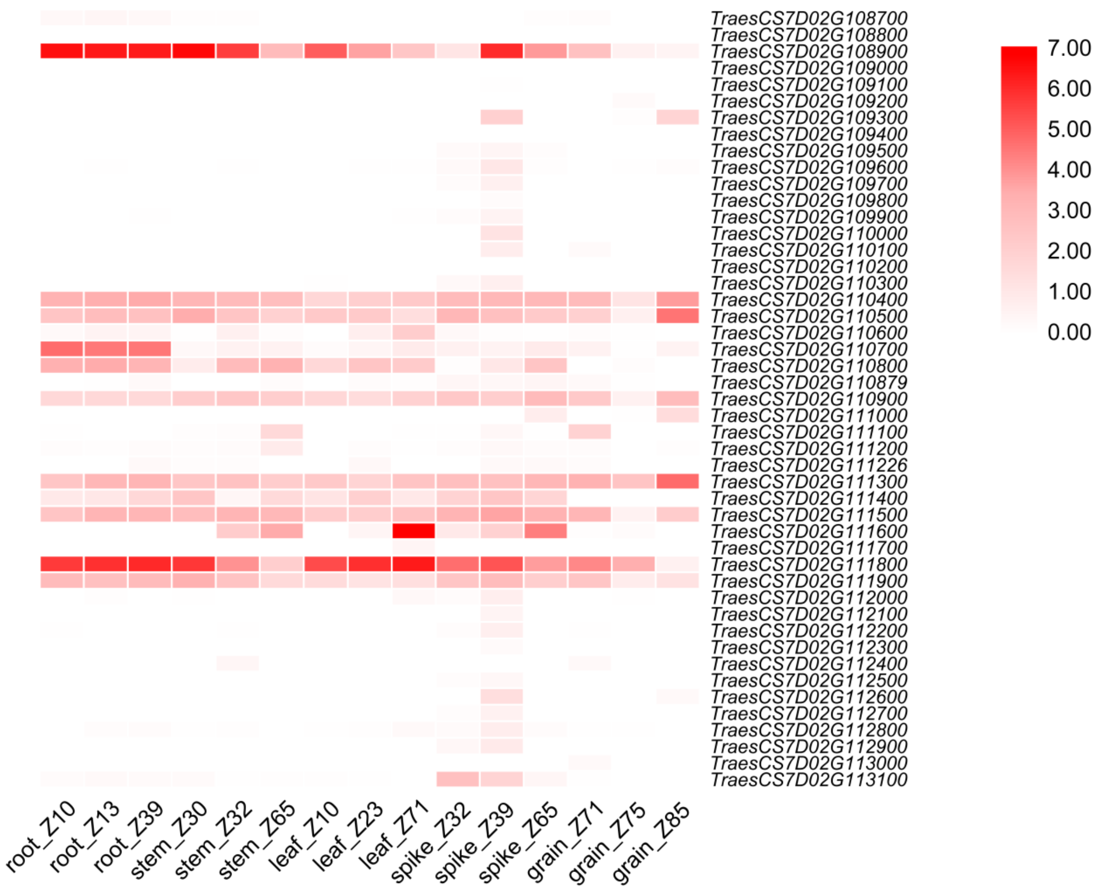

Supplementary Fig. S3 Heatmap showing the expression profile of DEGs at 15 development stages

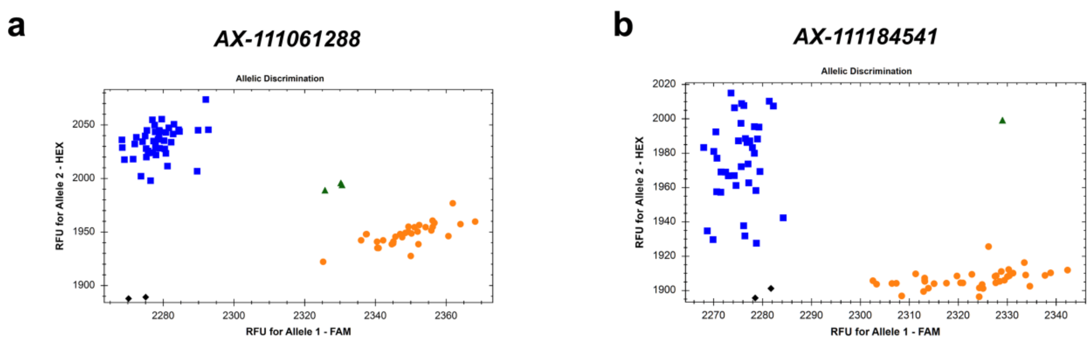

Supplementary Fig. S4 Allelic segregation of KASP markers *AX-111061288* (a) and *AX-111184541* (b) for *QTkw.cas-7D.2* and *QKw.cas-7D*.
